# Nutraceutical pill containing berberine versus ezetimibe on plasma lipid pattern in hypercholesterolemic subjects and its additive effect in patients with familial hypercholesterolemia on stable cholesterol-lowering treatment

**DOI:** 10.1186/1476-511X-11-123

**Published:** 2012-09-22

**Authors:** Livia Pisciotta, Antonella Bellocchio, Stefano Bertolini

**Affiliations:** 1 Department of Internal Medicine, University of Genoa, Viale Benedetto XV n. 6, 16132, Genoa, Italy

**Keywords:** Berberine, Ezetimibe, Primary polygenic hypercholesterolemia, Familial Hypercholesterolemia, PCSK9

## Abstract

**Background:**

Although statins (STs) are drugs of first choice in hypercholesterolemic patients, especially in those at high cardiovascular risk, some of them are intolerant to STs or refuse treatment with these drugs. In view of this, we have evaluated the lipid-lowering effect of a nutraceutical pill containing berberine (BBR) and of ezetimibe, as alternative treatments, in monotherapy or in combination, in 228 subjects with primary hypercholesterolemia (HCH), with history of STs intolerance or refusing STs treatment. In addition, since PCSK9 was found up-regulated by STs dampening their effect through an LDL receptors (LDLRs) degradation, and BBR suppressed PCSK9 expression in cellular studies, we supplemented the stable lipid-lowering therapy of 30 genotype-confirmed Familial Hypercholesterolemia heterozygotes (HeFH) with BBR, searching for a further plasma cholesterol reduction. Plasma lipid pattern was evaluated at baseline and during treatments.

**Results:**

In HCH subjects the nutraceutical pill resulted more effective than EZE in lowering LDL cholesterol (−31.7% vs −25.4%, P < 0.001) and better tolerated. On treatment, LDL-C level below 3.36 mmol/L (≤130 mg/dl) was observed in 28.9% of subjects treated with the nutraceutical pill and 11.8% of those treated with EZE (P <0.007). In the group treated with EZE the subjects carrying the G allele of the g.1679 C ***>*** G silent polymorphism of *NPC1L1* gene showed a higher response to EZE than homozygous for the common allele (GG + CG: LDL-C −29.4±5.0%, CC −23.6±6.5%, P <0.001). Combined treatment with these drugs was as effective as STs in moderate doses (LDL cholesterol −37%, triglycerides −23%). In HeFH patients the addition of BBR resulted in LDL cholesterol reductions inversely related to those induced by the stable therapy (r = −0.617, P <0.0001), with mean 10.5% further decrease.

**Conclusions:**

The alternative treatments tested in our HCH subjects were rather effective and safe. The findings in HeFH patients suggest that BBR might act in vivo increasing expression and stability of LDLRs and/or suppressing PCSK9 expression.

## Background

An elevated level of LDL cholesterol (LDL-C) in plasma is the major risk factor for cardiovascular disease (CVD) [1]. The HMG-CoA reductase inhibitors (Statins, STs) are the drugs of first choice to lower plasma cholesterol, especially in patients at high or very high risk for cardiovascular disease (CVD), in view of their documented and dose-related efficacy in reducing CVD morbidity and mortality in both primary and secondary prevention [2-5]. However, 10-15% of patients result to be intolerant to any ST, even at low daily doses, for muscle side effects, ranging from mild myalgia without increase of creatine kinase (CK) to more severe muscle symptoms with significant CK elevations [6,7], and 1-3% show an asymptomatic elevation of transaminases (AST, ALT) in the absence of clear hepatic toxicity [6]. Some other patients, in primary prevention, refuse STs treatment since they are worried about possible side effects. Alternative hypocholesterolemic treatments to be considered for the above mentioned cases include plant sterols and stanols, the cholesterol absorption inhibitor ezetimibe, bile acid sequestrants (especially the well tolerated colesevelam) and some nutraceutical products containing various associations of red yeast rice, policosanols, phytosterols and berberine [2,6-8].

When monotherapy results in unsatisfactory lipid-lowering effect, combined treatment with some of these products can be prescribed [9].

The aims of this paper were: i) to evaluate the plasma lipid-lowering effect of a nutraceutical product containing berberine, policosanols and red yeast rice (BBR/P/RR), in comparison to that induced by ezetimibe (EZE) as monotherapy in subjects with primary polygenic hypercholesterolemia (HCH) intolerant to STs or refusing treatment with these drugs; ii) to test the effect of the combined therapy with BBR/P/RR and EZE on plasma lipid concentrations in a subgroup of these subjects, low responders to monotherapy and iii) to search for an additional plasma cholesterol reduction induced by the supplementation with BBR/P/RR in a small number of genotype-confirmed patients with heterozygous Familial Hypercholesterolemia (HeFH), who were on stable-dose treatment with STs or STs plus EZE, in view of the effect of Proprotein Convertase Subtilisin/Kexin type 9 (PCSK9) on low-density lipoprotein receptor (LDLR) degradation, the increase of plasma PCSK9 level induced by STs [10] and the inhibitory effect of BBR on PCSK9 expression found in human-derived cultured cells [11].

## Methods

### Subjects

#### Comparison between the lipid-lowering effect of a nutraceutical-combined pill containing berberine (BBR/P/RR) and of ezetimibe (EZE) in monotherapy

Among outpatients attending our Lipid Clinic we considered eligible for this study subjects with primary polygenic HCH, diagnosed on the basis of personal and family studies, who had a negative history of cardiovascular disease and were previously found to be intolerant to STs or were refusing treatment with these drugs. Cardiovascular evaluation was performed in each subject by ECG stress testing and ultrasound examination of the carotid arteries. The patients with secondary forms of hyperlipidaemia, including type 2 diabetics, subjects with monogenic forms of HCH and those with silent ischemia at ECG stress test and/or arterial carotid atherosclerosis with more than 40% stenosis or soft plaque morphology were excluded from the study.

After a three-month run-in period with a plasma lipids stabilizing diet (poor in saturated fats and cholesterol), 270 subjects were randomized by lot on 2:1 basis either to a treatment with a nutraceutical-combined pill (containing berberine 500 mg, policosanol 10 mg and red yeast rice 200 mg; BBR/P/RR) or ezetimibe 10 mg/day (EZE) for six months.

Plasma lipid concentrations were assessed after diet (baseline condition) and every two months during treatment, and the mean value of the three determinations of each lipid parameter was considered for the statistical comparison. Laboratory analysis was performed “double-blind” because laboratory operators did not know the assigned treatment.

Seventy-six subjects on EZE (31 males and 45 females) completed the six-month follow-up period; eight subjects were excluded for poor compliance and six left the study for gastrointestinal or biochemical side-effects (AST/ALT and/or CK elevation). For comparison purposes, each of the 76 subjects on EZE was matched for gender, age and baseline cholesterol level with two subjects on BBR/P/RR treatment (Table 1). No significant side-effects were recorded in the group of subjects receiving the nutraceutical pill.

**Table 1:** Clinical features and baseline plasma lipid concentrations of the two groups of subjects with primary hypercholesterolemia (HCH) treated with BBR/P/RR or EZE for six months

	**BBR/P/RR**	**EZE**	**P**
Males/Females	62/90	31/45	
Age (years)	57.3 ± 12.1	58.3 ± 12.3	NS
BMI (kg/m^2^)	23.9 ± 2.9	23.5 ± 2.8	NS
Arterial hypertension	30.2%	31.5%	NS
Carotid atherosclerosis^*^	26.3, 48.0, 3.9%	31.5, 51.3, 3.9%	NS
TC (mmol/L)	7.63 ± 0.50	7.72 ± 0.49	NS
LDL-C (mmo/L)	5.36 ± 0.48	5.36 ± 0.52	NS
HDL-C (mmo/L)	1.55 ± 0.35	1.57 ± 0.34	NS
non-HDL-C (mmol/L)	6.07 ± 0.54	6.15 ± 0.55	NS
TG (mmol/L)^†^	1.46 (1.22-1.90)	1.67 (1.24-2.12)	NS

^*^Percent of subjects with increased intima-media thickness, with fibrous-calcific plaques with less than 30% stenosis and with fibrous-calcific plaques with 30-40% stenosis, respectively. ^†^Median (interquartile range). NS: not significant.

#### Combined lipid-lowering effect of BBR/P/RR and EZE

As an extension of the former study, 26 subjects (57.7 ± 9.0 years of age, BMI 23.3 ± 3.3 kg/m^2^), 12 (5 males and 7 females) on BBR/P/RR and 14 (7 males and 7 females) on EZE as monotherapy, received combined treatment with the two products for a further three-month period. These subjects were selected from those who, during monotherapy, had a percent decrease of LDL-C below the median level (−29%) of the distribution of the values obtained in the whole group of subjects on mono-therapy (n. 152 on BBR/P/RR and n.76 on EZE), and gave their consent to combined treatment. Plasma lipid concentrations were determined monthly and for each lipid parameter the mean value of the three measures was compared with the mean value obtained during mono-therapy.

#### Evaluation of the effect on plasma lipid concentrations of BBR/P/RR supplementation in patients with heterozygous Familial Hypercholesterolemia (HeFH) on stable-dose treatment with statins (STs) or statins plus ezetimibe (STs + EZE)

Thirty patients with HeFH previously genetically characterized in our laboratory (21 males and 9 females, 56.5 ± 9.2 years of age, 12 with receptor-defective (RD) and 18 with receptor-negative (RN) *LDLR* gene mutations [12]), who were on stable-dose treatment with the maximal tolerated dose of STs or STs plus EZE for one year or more [see Additional file 1: Table S1], received BBR/P/RR pill for three months as supplementary therapy. Plasma lipid concentrations were determined monthly and for each lipid parameter the mean value of the three measures was compared with the mean value obtained during STs or STs plus EZE therapy.

Written informed consent was obtained from each participant and the study protocol was approved by the local institutional human investigation committee.

### Biochemical analysis

Plasma concentrations of total cholesterol (TC) and triglycerides (TG) were measured by standardized enzymatic methods. High-density lipoprotein cholesterol (HDL-C) was measured in plasma supernatant after precipitation of apoB-containing lipoproteins by phosphotungstate-MgCl_2_. Low-density lipoprotein cholesterol (LDL-C) was calculated by the Friedewald's formula. Serum non-HDL-C concentrations were calculated by subtracting HDL-C concentrations from TC concentrations.

### Genotyping for the g.1679 C > G polymorphism of NPC1L1 gene

Genomic DNA was extracted from peripheral blood leukocytes using standard method. The screening for the silent polymorphism g.1679 C ***>*** G (c.816 C ***>*** G, L272L) of *NPC1L1* gene [GenBank: NG_013088.1] was performed by amplification of the central portion of exon 2 using the following primers: 5’-CCA GCT AGG GTC TGG ACA ACT CC -3’ (forward) and 5’-GGA TGA CAG ATA GCA CCA AGA TGG -3’ (reverse). Since the presence of G allele eliminates a Taq I restriction site (T/CGA), the PCR product was incubated with 10 U of Taq I (New England Biolabs, Beverly, MA, USA) at 65°C for 1 h and the digestion products (387 and 269 bp for the C allele and 656 bp for the G allele) were separated on 2% agarose gel.

### Statistical analyses

The statistical analyses were performed using PASW 18.0 statistical software package (SPSS Inc., Chicago, IL). Data are presented as mean ± SD for continuous variables, medians and interquartile ranges for triglycerides which have a skewed distribution. Differences in the distribution of categorical variables were assessed by *χ*^2^ with Yates’s correction or Fisher's exact tests. Triglyceride values, which were not normally distributed, were logarithmically transformed before analysis. The statistical significance of the differences between baseline and treatment lipid values, as well as between monotherapy (BBR/P/RR or EZE) and combined treatment (BBR/P/RR plus EZE), was assessed by Student's *t*-test for paired data or Wilcoxon test. The significance of the differences between treatments (BBR/P/RR vs EZE) was evaluated comparing percent variations of the lipid parameters by Student’s *t*-test or Mann–Whitney test, as appropriate. In patients with HeFH, on stable-dose treatment with STs or STs plus EZE, the effect of additional therapy with BBR/P/RR was assessed by ANOVA and multiple comparisons among pairs of means were performed by *t*-tests with Bonferroni's correction. Percent variations induced by STs or STs plus EZE were compared with those induced by BBR/P/RR supplementation by Student’s *t*-test for paired data or Wilcoxon test. The correlation between changes induced by STs or STs plus EZE and those induced by BBR/P/RR supplementation was evaluated by Pearson’s and Spearman’s tests.

## Results

### Comparison between the lipid-lowering effect of a nutraceutical-combined pill containing berberine (BBR/P/RR) and of ezetimibe (EZE) in monotherapy

**Figure 1:**
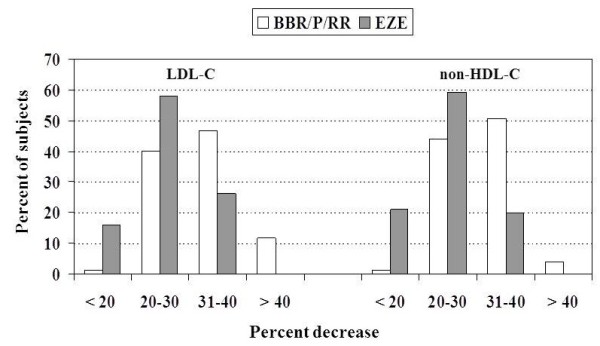
Percent distribution of subjects treated with BBR/P/RR or EZE according to the percent decreases of LDL-C and non-HDL-C.

Table 2 shows the effects on lipid parameters respectively induced by the treatment with BBR/P/RR and EZE in the two groups of subjects with primary HCH. The comparison of the two treatments in terms of percent lipid variations showed that BBR/P/RR was more effective than EZE in reducing TC, LDL-C and non-HDL-C levels, and showed a tendency to lower TG more than EZE. The percentage of subjects with more than 30% decrease of LDL-C and non-HDL-C resulted to be higher in the group treated with BBR/P/RR than in the group treated with EZE (P <0.0001) (Figure 1). No significant effect on HDL-C level was recorded with either treatment, although a tendency to increase was observed in patients treated with EZE. On treatment, LDL-C level below 3.36 mmol/L (≤130 mg/dl) was observed in 28.9% of subjects treated with BBR/P/RR and 11.8% of those treated with EZE (P <0.007); non-HDL-C level below 4.14 mmol/L (≤160 mg/dl) was observed in 44.0% of subjects treated with BBR/P/RR and in 21.0% of those treated with EZE (P <0.002). A DNA sample was available for 70 out of 76 subjects treated with EZE. These subjects were genotyped for the g.1679 C ***>*** G silent polymorphism of the *NPC1L1* gene, which had previously been found to influence the cholesterol-lowering effect of EZE [13,14]. Indeed, the carriers of the G allele (3 GG and 23 CG) showed a higher response to EZE than homozygous for the common allele (44 CC) (LDL-C −29.4±5.0% vs −23.6±6.5%, P <0.001). 

**Table 2:** Comparison between treatments with BBR/P/RR and EZE on plasma lipid concentrations in HCH patients. Six months follow-up

	**BBR/P/RR**			**EZE**			^**†**^**P**
**Plasma lipids (mmo/L)**	**Baseline**	**On treatment**	**Percent change**	**Baseline**	**On treatment**	**Percent change**	
TC	7.63 ± 0.50	5.77 ± 0.49^*^	−24.2 ± 5.2	7.72 ± 0.49	6.25 ± 0.51^*^	−19.0 ± 4.6	< 0.001
LDL-C	5.36 ± 0.48	3.66 ± 0.48^*^	−31.7 ± 7.0	5.36 ± 0.52	4.00 ± 0.52^*^	−25.4 ± 6.4	< 0.001
HDL-C	1.55 ± 0.35	1.54 ± 0.37	−0.64 ± 7.2	1.57 ± 0.34	1.58 ± 0.35	+1.24 ± 6.9	NS
non-HDL-C	6.07 ± 0.54	4.22 ± 0.53^*^	−30.3 ± 6.5	6.15 ± 0.55	4.66 ± 0.58^*^	−24.2 ± 5.9	< 0.001
TG	1.46 (1.22-1.90)	1.12 (0.95-1.41)^*^	−19.5 ± 16.1	1.67 (1.24-2.12)	1.43 (1.08-1.80)^*^	−14.9 ± 11.5	NS

Data are means ± SD or medians (interquartile ranges). ^*^P < 0.001 vs baseline values (Student’s *t* test for paired data or Wilcoxon test); ^†^P: significance of the differences between percent changes induced by BBR/P/RR and by EZE treatments (Student’s *t* test and Mann–Whitney test, respectively). NS: not significant.

### Combined lipid-lowering effect of BBR/P/RR and EZE

Table 3 shows the lipid-lowering effect of the combined therapy with BBR/P/RR and EZE in 26 subjects low-responders to monotherapy. In comparison to monotherapy, the combined treatment caused a further mean decrease of TC, LDL-C, non-HDL-C and TG of −9.7%, -13.6%, –12.5% (P <0.001) and −4.5% (P <0.005), respectively. During combined treatment 50% of the subjects reached an LDL-C level below 3.36 mmol/L (≤130 mg/dl) and 65.4% a non-HDL-C below 4.14 mmol/L (≤160 mg/dl). This combination was well tolerated and did not induce side effects.

**Table 3:** Comparison between plasma lipid changes induced by monotherapy with BBR/P/RR (n. 14 subjects) or EZE (n. 12 subjects) and combined therapy with BBR/P/RR plus EZE in HCH patients

**Lipid parameter**	**Baseline**	**Monotherapy**	**% changes**	**Combined therapy**	**% changes**	^**‡**^**P**
TC (mmol/L)	7.74 ± 0.40	6.34 ± 0.40^*^	−18.0 ± 3.5	5.59 ± 0.40^†^	−27.7 ± 3.8	< 0.001
LDL-C (mmol/L)	5.47 ± 0.51	4.19 ± 0.48^*^	−23.5 ± 3.5	3.44 ± 0.44^†^	−37.1 ± 4.5	< 0.001
HDL-C (mmol/L)	1.58 ± 0.40	1.59 ± 0.42	+0.17 ± 8.2	1.60 ± 0.42	+1.10 ± 5.7	NS
non-HDL-C (mmol/L)	6.15 ± 0.59	4.75 ± 0.57^*^	−22.8 ± 3.8	3.98 ± 0.49^†^	−35.3 ± 4.2	< 0.001
TG (mmol/L)	1.48 (1.08-1.89)	1.12 (0.95-1.47)^*^	−18.6 ± 15.5	1.09 (0.94-1.35)^†^	−23.1 ± 12.9	< 0.005

Values are means ± SD or medians (interquartile ranges). ^*^P <0.001 vs baseline values and ^†^P <0.001 vs monotherapy; ^‡^P: significance of the differences between percent changes induced by monotherapy and by combined therapy (Student’s *t* test for paired data or Wilcoxon test, respectively). NS: not significant.

### Supplementary therapy with BBR/P/RR in HeFH patients on stable-dose treatment with STs or STs + EZE

Table 4 shows plasma lipid concentrations at baseline, during treatment with a stable dose of STs or STs plus EZE and after BBR/P/RR supplementation in 30 HeFH patients attending our Lipid Clinic. Tendon xanthomatosis, coronary artery disease and previous coronary revascularization were present in 17%, 33% and 25% of HeFH-RD patients and in 89%, 61% and 33% of HeFH-RN patients, respectively.

**Table 4:** Plasma lipid changes induced by supplementary treatment with BBR/P/RR in 30 HeFH patients on stable-dose therapy with STs or STs plus EZE

**Lipid parameter**	**Baseline**	**on STs or STs** + **EZE**	**% changes vs baseline**	**plus BBR/P/RR**	**% changes after BBR/P/RR addition**	**P**
TC (mmol/L)	9.94 ± 1.49	6.35 ± 0.99^†^	−35.5 ± 8.5	5.54 ± 0.77^†‡^	−43.7 ± 7.3	< 0.001
LDL-C (mmol/L)	7.91 ± 1.35	4.47 ± 0.94^†^	−42.6 ± 10.9	3.65 ± 0.71^†§^	−53.2 ± 8.7	< 0.001
HDL-C (mmol/L)	1.29 ± 0.39	1.30 ± 0.35	+2.6 ± 10.3	1.33 ± 0.38	+4.3 ± 11.4	NS
non-HDL-C (mmol/L)	8.65 ± 1.47	5.05 ± 1.06^†^	−41.2 ± 9.8	4.20 ± 0.84^†‡^	−50.9 ± 8.7	< 0.001
TG (mmol/L)	1.48 (0.98-2.30)	1.27 (0.89-2.03)	−17.7 ± 17.2	1.11 (0.83-1.63)^*^	−23.1 ± 21.2	< 0.03

Values are means ± SD or medians (interquartile ranges). ^*^P <0.05 vs baseline, ^†^P < 0.02 vs baseline; ^‡^P < 0.05 vs STs and STs + EZE, ^§^P < 0.02 vs STs and STs + EZE (ANOVA and multiple comparisons among pairs of means by *t*-tests with Bonferroni's correction). ^¶^P: significance of the differences between percent changes induced by STs or STs + EZE and those induced by treatment supplementation with BBR/P/RR (Student’s *t* test for paired data or Wilcoxon test). NS: not significant.

**Figure 2:**
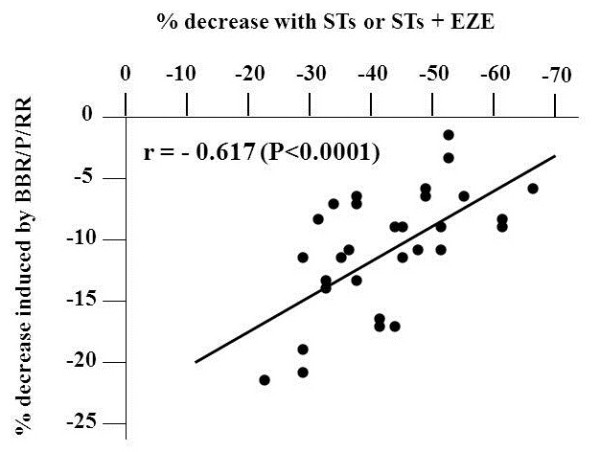
Pearson’s correlation between percent decrease of LDL-C induced by STs or STs/EZE and the additional percent decrease due to BBR/P/RR supplementation in genotype-confirmed HeFH patients.

On average BBR/P/RR significantly further decreased TC, LDL-C, non-HDL-C and TG levels by 8.1%, 10.5%, 9.7% and 5.4%, respectively. These effects were not significantly different between HeFH-RD and HeFH-RN patients. The additional percent decrease of plasma LDL-C induced by BBR/P/RR (calculated as the difference between percent decrease induced by treatment with STs or STs + EZE supplemented with BBR/P/RR minus that induced by STs or STs + EZE) showed a wide inter-individual variability (from −22.5% to −2.0%). Fifteen patients, showing more than 10% decrease of LDL-C level with respect to percent decrease observed during STs or STs + EZE therapy, were considered good-responders to BBR/P/RR treatment, while two patients, showing less than 5% decrease, were considered non-responders. During therapy with STs or STs + EZE an LDL-C value below 3.36 mmol/L (≤130 mg/dl) and non-HDL-C below 4.14 mmol/L (≤160 mg/dl) was reached only in 3 and 6 patients, respectively; after the addition of BBR/P/RR the patients reaching these levels were 11 and 14, respectively. In order to define whether the magnitude of the response to BBR/P/RR was affected by the pre-existing treatment with STs or STs + EZE, we plotted the percentage response to STs or STs + EZE against the additional LDL-C reduction (given as percentage) induced by BBR/P/RR. We observed a highly significant inverse correlation between these parameters with both Pearson’s and Spearman’s tests (r = −0.617 and r = −0.611, respectively; P <0.0001) (Figure 2).

## Discussion

Twenty-two percent of the subjects selected for treatment with BBR/P/RR or EZE had a previous history of intolerance to any STs, even at low dosage, while the others refused STs treatment for fear of possible side effects. Since at present there are no clinical data on the reduction of CVD events with these treatments, we enrolled for this study only subjects with primary HCH and moderate CVD risk. The BBR/P/RR combination resulted more effective than EZE, in terms of reduction of TC, LDL-C and non-HDL-C (Figure 1); neither of the treatments significantly affected HDL-C concentrations, although a small increase was observed with EZE, but both reduced TG levels to the same extent (Table 2). Moreover, BBR/P/RR pill resulted to be better tolerated than EZE monotherapy. Previous studies, placebo or dietary regimen controlled, which evaluated the plasma lipid-lowering effects of this nutraceutical pill in groups of various sizes of middle-aged or elderly subjects, with moderate forms of mixed hyperlipidemia, had given variable results (decrease of LDL-C from −10.8% to −31% and TG from −9.5% to 26%) [15-18]. In our group of subjects with a more severe form of primary HCH treated with BBR/P/RR for six months, LDL-C, non-HDL-C and TG were reduced by 32%, 30% and 19.5%, respectively. Discrepancy with some of the previous findings may be due to several factors, including the type of hyperlipidemia (isolated more severe HCH versus mixed hyperlipidemia), difference in body weight and glucose tolerance, compliance to medication and concomitant therapy with drugs interfering with lipoprotein metabolism.

In relation to the composition of the nutraceutical pill, the mechanisms underlying its lipid-lowering effect must be considered for each single component.

Chinese red yeast rice extract is a medicinal agent made by fermenting the yeast, Monascus purpureus, over rice. Monascus yeast produces a family of substances called monacolins, including monacolin K (also called mevilonin or lovastatin), capable of inhibiting the enzyme HMG-CoA reductase, and it also contains monounsaturated fatty acids, isoflavones and phytosterols, all capable of lowering LDL-C. Several studies, including one meta-analysis, all support the beneficial effect of red yeast rice on blood lipid profile in hyperlipidemic subjects [19] and this effect was confirmed in patients intolerant to STs, where red yeast rice preparations induced a dose-dependent decrease of LDL-C, from 21% to 30%, without serious adverse effects in most of the cases [20,21]. However, in all these studies red yeast rice preparations, containing a variable amount of monacolin K and other monacolins per fixed dose of red yeast rice [22], were used in daily doses, ranging from 0.6 to 4.8 g, much higher than that present in the nutraceutical pill administered in this study (200 mg with standardized content of 3 mg monacolin K), accounting for some reports of myopathy in patients treated with high doses of red yeast rice.

Policosanol is a mixture of long-chain primary alcohols isolated from sugar cane wax. Apart from early clinical studies conducted by Cuban groups showing beneficial effects of the policosanol on the plasma lipid profile [19], more recent randomized controlled trials in hyperlipidemic Caucasian subjects did not find any lipid-lowering effect of this compound either in monotherapy in doses ranging from 10 to 80 mg/day [23] or in combination with STs [24]. On the basis of these findings, we believe that the 10 mg of policosanol in our nutraceutical pill do not contribute to its hypolipidemic effect.

The lipid-lowering effect of BBR, an isoquinoline alkaloid extracted from many herbal plants (Rhizoma coptidis, Hydrastis Canadensis, Berberis vulgaris), was reported for the first time in Chinese patients with type IIa or IIb hyperlipidemia treated with BBR alone (500 mg twice per day). In that study BBR decreased TC, LDL-C and TG by 20%, 25% and 8% in type IIa and by 29.5%, 11% and 48% in type IIb, respectively [25]. Studies in animals and cultured cells had clarified the mechanisms underlying the effects of this drug. BBR increases hepatic LDLR mRNA and LDLR protein stabilizing mRNA by post-transcriptional mechanism, which involves the activation of the extracellular signal-regulated kinase (ERK) signaling pathway [25]. The activation of ERK inhibits the expression of hnRNP I (heterogeneous nuclear ribonucleo-protein I) and KSRP (KH-type splicing regulatory protein), which, interacting with cis-regulatory sequences (AU-rich elements) in the proximal section of the 3’ untranslated region (UTR) of LDLR mRNA, reduce its stability [26]. By the activation of the JNK/c-jun pathway, BBR also increases the transcriptional activity of *LDLR* promoter [27]. In addition, BBR may reduce plasma lipids inhibiting hepatic cholesterol and triglyceride synthesis through the activation of AMP-activated protein kinase (AMPK), which inactivates HMG-CoA reductase and Acetyl-CoA carboxylase enzymes [28].

In the original study of hypercholesterolemic type IIa patients, the 25% decrease of LDL-C levels was obtained with a dose of BBR two-fold that present in our nutraceutical pill [25]. Moreover, Cicero et al. [15] in their hyperlipidemic patients, using 500 mg/day of BBR alone, observed only a 20% decrease in LDL-C. In view of these findings and of the results obtained in the present study with low dose of both red yeast rice and BBR, we can suppose a synergistic effect of these two components on hepatic lipids synthesis inhibition, in addition to the BBR-mediated increase of LDLR mRNA and LDLR protein abundance.

EZE inhibits the absorption of dietary and biliary cholesterol, as well as of plant sterols, at the brush border of jejunal enterocytes, and the re-absorption of the biliary cholesterol by the liver cells, selectively blocking the activity of NPC1L1 transporter in enterocytes and canalicular membranes of hepatocytes [29]. By reducing the amount of chylomicron cholesterol supplied to the liver, EZE decreases hepatic cholesterol content, leading to increased LDLRs expression, which results in an increased uptake of LDL from the plasma and decreased circulating LDL-C levels [30]. However, the effect of EZE on LDLRs expression is partially counteracted by an increase in cholesterol synthesis, which is suppressed by STs [31]. This explains the additive effect on LDL-C lowering of EZE and STs when these drugs were co-administered [32].

From a meta-analysis of eight randomized controlled trials [33] and from 14 more recent studies, which as a whole investigated 4110 hyperlipidemic subjects (LDL-C 4.32±0.49 and TG 1.90±0.75 mmol/L) treated for 12 weeks, it results that monotherapy with 10 mg/day of EZE decreases LDL-C and TG by 20.4% and 8,9%, respectively, and increase HDL-C by 3%. In subjects we treated with EZE, although in a context of a large inter-individual variability (decrease in LDL-C from −9.2% to −38.8%), we unexpectedly observed a higher mean percent reduction of plasma LDL-C (−25.4%). Possible explanations for these results, in comparison with those reported above, were the higher baseline levels of LDL-C [34] of our patients, a more prolonged treatment and the genetic background of our population, with reference to some single nucleotide polymorphisms or non-synonymous sequence variants of *NPC1L1* gene associated with a higher LDL-C lowering response to EZE [14]. However, this last assumption must be considered with caution, since in the present paper we have investigated only one genetic variant and have found a frequency of the minor allele similar to that detected in other populations [13].

In the small subgroup of our subjects, who were considered relatively low responders to monotherapy, the combined treatment with BBR/P/RR and EZE decreased LDL-C by 37%, non-HDL-C by 35% and TG by 23% (Table 3), owing to the inhibition of cholesterol absorption associated with a suppression of hepatic lipid synthesis, causing a more sustained expression of LDLRs. The effects of the combined treatment were similar to those induced by simvastatin 20 mg/day, atorvastatin 10 mg/day and rosuvastatin 5 mg/day and higher than those obtained with pravastatin 40 mg/day in patients with primary HCH [35], suggesting a satisfactory alternative treatment of patients intolerant to STs.

In our group of genotype-confirmed HeFH patients, who were on stable treatment with STs or STs/EZE combination (see Additional file 1: Table S1), the supplementation of the treatment with the nutraceutical pill containing BBR, although in the context of a large inter-individual variability, induced a mean 10.5% further decrease of plasma LDL-C level [Table 4. The mechanisms underlying this effect are likely to be BBR-mediated and might consist in: i) an increased expression of LDLRs [27] encoded by the wild type allele, coupled with their prolonged half-life [25,26] and/or ii) a reduced expression of PCSK9 [11] and consequently a lower LDLRs degradation (see below).

PCSK9 is a serine protease expressed in the liver and intestine, which after auto-cleavage in endoplasmic reticulum (ER) moves to the cell surface and is secreted into the plasma [10,36]. Circulating PCSK9 binds to LDLR on the cell surface of hepatocytes, the LDLR/PCSK9 complex is internalized into the cell and in lysosomes LDLR undergo a PCSK9-mediated degradation. PCSK9, independently from endocytosis, can also bind LDLRs within the cell targeting them for degradation in lysosomes [36]. The role of PCSK9 as post-transcriptional regulator of the amount of LDLRs in the liver is supported by the finding that gain of function mutations of PCSK9 in humans cause hypercholesterolemia, while loss of function mutations cause hypocholesterolemia, and respectively increase and reduce cardiovascular risk [10,36]. The promoter region of *PCSK9* gene contains the HNF1 and SRE sites, which respectively bind the transactivation proteins Hepatocyte Nuclear Factor 1α (HNF1α) and Sterol Regulatory Element Binding Protein 2 (SREBP2). HNF1α is essential for *PCSK9* basal transcription and for SREBP2-induced maximal gene expression in response to intracellular cholesterol depletion [37].

Treatment with STs, by inhibiting cholesterol synthesis, causes cholesterol depletion in the liver and consequently the translocation of SREBP2 from cytoplasm to the nucleus where this transcriptional protein simultaneously up-regulates the expression of LDLR and PCSK9; the latter, by increasing LDL receptors degradation, may reduce the LDL-C lowering effect of STs. In vitro studies and some clinical observations support this mechanism. Dubuc et al. [38] in HepG2 cells and in human primary hepatocytes found that STs upregulated the expression of PCSK9 mRNA and that this induction was dose-dependent and reversed by mevalonate. Clinical investigations in HCH patients showed a dose-dependent increase of PCSK9 in plasma during treatment with atorvastatin or rosuvastatin [39,40]. The treatment with STs/EZE combination, through a more pronounced depletion in hepatic cholesterol content, induced an increase in plasma PCSK9 more pronounced (~77%) than STs alone in the same doses (~45%) [39]. These results provide one possible explanation why increasing doses of STs fail to achieve a proportional LDL-C reduction (only about 6% with each doubling of the dose), in view of the dose-dependent increase of PCSK9 expression induced by STs and the consequent PCSK9-mediated degradation of LDLRs [40].

Starting from the finding that ST administration to *Pcsk9* knockout mice produced an exaggerated increase in LDLRs in liver and enhanced LDL clearance from their plasma [41], and from the observation that HeFH patients, who also carried loss of function mutations of PCSK9, showed a greater than expected reduction of LDL-C levels during STs therapy [42], novel strategies have been adopted in experimental animal models to inhibit or reduce PCSK9 production or activity; this provides the background for future therapies aimed to increase the LDL-C lowering effect of the currently used pharmacological treatments. These strategies include the PCSK9 gene silencing with modified antisense oligonucletides or short interfering RNA (siRNAs) directed against the PCSK9 mRNA, monoclonal antibodies directed against circulating PCSK9 protein which inhibit its binding to LDLR and small peptides which prevent PCSK9 maturation or its interaction with LDLR [43]. The results till now obtained have showed that the inhibition of PCSK9 resulted in an increase of hepatic LDLRs and in a significant reduction of LDL-C levels, and in a strong additional decrease of LDL-C in animals receiving STs.

In vitro studies in human hepatoma-derived cell lines (HepG2 and Huh7 cells) showed that BBR decrease PCSK9 mRNA and protein levels in a time- and dose-dependent manner [11,44]. BBR acts inducing a coordinate reduction of the cellular amount of HNF1α and SREBP2 proteins, and consequently reduces the binding of these proteins, which synergistically transactivate the *PCSK9* gene transcription to the respective HNF1 and SRE-1 sequences in the promoter; this mechanism results in a strong suppression of PCSK9 production [37]. Moreover, BBR counteracts the stimulating effect of STs on PCSK9 transcription and increases LDLR mRNA and LDLR-protein levels [11,37,44]. A previous study in hyperlipidemic rats showed that the combination of BBR with simvastatin at low dosage caused a decrease in plasma LDL-C and TG significantly higher than monotherapy with either of these drugs and similar to that induced by a double dose of simvastatin alone, and that the combined treatment up-regulated the LDLR mRNA in rat livers to a level 1.6-fold higher than the monotherapies did. These results were confirmed in Chinese patients with moderate HCH treated with simvastatin 20 mg/day plus BBR 1 g/day or with either of these drugs alone; in these patients the combination lowered LDL-C and TG to extents much higher than monotherapy [45]. These findings suggest that BBR might strengthen in vivo the reduction of plasma LDL-C induced by the treatment with STs.

On these bases we tested the effect of BBR supplementation in our HeFH patients. Unfortunately, at the time of data collection we were unable to measure plasma PCSK9 concentrations before and after BBR supplementation and consequently we can not prove that the further LDL-C reduction obtained with BBR was really mediated by an inhibition of PCSK9 expression. However, in these HeFH patients we observed an inverse correlation between the reduction of LDL-C levels obtained with STs or STs + EZE and the additional decreases induced by BBR (Figure 2). Considering that previous studies found a direct relationship between the reductions of LDL-C levels induced by STs and the increases of PCSK9 in plasma [39,46], our finding of the above mentioned inverse correlation might provide an indirect proof of the BBR-mediated inhibition of PCSK9. Indeed, in the presence of progressively higher levels of PCSK9, we expect the inhibitory effect of the fixed dose of BBR on PCSK9 production to be progressively reduced, accounting for a proportional reduction of the additional LDL-C lowering effect. However, concomitant or different mechanisms, such as an increased expression of LDLRs, coupled with their prolonged half-life, may explain our findings.

## Conclusions

Among the available alternative lipid-lowering treatments of subjects with primary HCH intolerant to STs or refusing therapy with these drugs, we have evaluated the effects of a nutraceutical pill containing BBR and red yeast rice and of EZE and have showed the greater efficacy of the former product. Moreover, with their combination we observed a decrease in plasma LDL-C and TG comparable to that induced by the administration of moderate doses of STs. In patients with HeFH on stable treatment with LDL-C lowering validated drugs, the supplementation with the nutraceutical pill resulted in a further decrease of LDL-C superior to that obtained doubling the dose of ST.

## Abbreviations

TC: Total cholesterol; LDL-C: Low-density lipoprotein cholesterol; HDL-C: High-density lipoprotein cholesterol; non-HDL-C: Non-high-density lipoprotein cholesterol; TG: Triglycerides; BBR: Berberine; P: Policosanol; RR: Red yeast rice; EZE: Ezetimibe; STs: Statins; CVD: Cardiovascular disease; HMG-CoA: Hydroxyl-methylglutaryl coenzyme A; LDLRs: LDL receptors; HCH: Hypercholesterolemia; HeFH: Heterozygous Familial Hypercholesterolemia; RD: Receptor defective; RN: Receptor negative; NPC1L1: Niemann-Pick C1-like 1; ERK: Extracellular signal-regulated kinase; hnRNP I: Heterogeneous nuclear ribonucleoprotein I; KSRP: KH-type splicing regulatory protein; JNK/c-jun: C-jun N-terminal kinase; AMPK: AMP-activated protein kinase; PCSK9: Proprotein convertase subtilisin/kexin type 9; HNF1α: Hepatocyte nuclear factor 1α; SREBP2: Sterol regulatory element binding protein 2.

## Competing interests

The authors have no conflict of interest to declare.

## Authors’ contributions

SB: design of the study, statistical analysis, writing of the paper LP: collecting clinical data, genetic analysis AB: biochemical and genetic analyses. All authors read and approved the final manuscript.

## Authors’ information

SB: MD, full professor of Internal Medicine, Department of Internal Medicine, University of Genoa, Italy. LP: MD, researcher of Internal Medicine, Department of Internal Medicine, University of Genoa, Italy. AB: PhD experimental and molecular pathology, Department of Internal Medicine, University of Genoa, Italy.

## Supplementary Material

Additional file 1 Table S1 Clinical and genetic characteristics of HeFH patients receiving supplementary treatment with BBR/P/RR. Click here for file
