# Supplementary material for: Nutraceutical pill containing berberine versus ezetimibe on plasma lipid pattern in hypercholesterolemic subjects and its additive effect in patients with familial hypercholesterolemia on stable cholesterol-lowering treatment
Source: Lipids Health Dis. 2012 Sep 22;11:123. doi: 10.1186/1476-511X-11-123 (PMC3477057; doi:10.1186/1476-511X-11-123)
Supplement: Additional file 1 Table S1 — Clinical and genetic characteristics of HeFH patients receiving supplementary treatment with BBR/P/RR. [file 1476-511X-11-123-S1.docx]

**Additional file 1**

Supplementary Table. Clinical and genetic characteristics of HeFH patients receiving supplementary treatment with BBR/P/RR.

| Patient | Sex | Age (y) | LDLR gene mutation | Clinical data | Current therapy |
| --- | --- | --- | --- | --- | --- |
| 1 | F | 64 | c.68 -?_1845 +?del (del Ex2-12) | Tx, silent ischemia, CA-ATS | S40/E10 |
| 2 | M | 67 | c.304C>T, p.Q102X | Tx, 3V-CAD, CABG at 41, PCI at 60 | A40/E10 |
| 3 | M | 60 | c.415G>A, p.D139N | Tx, MI at 41, CA-ATS | S40/E10 |
| 4 | F | 43 | c.415G>A, p.D139N | CA-ATS | S10/E10 |
| 5 | M | 64 | c.616_617insA, p.S206KfsX12 | Tx, MI at 44, CA-ATS | S60/E10 |
| 6 | M | 70 | c.662A>G, p.D221G | Tx, Ac, CA-ATS | R20 |
| 7 | M | 67 | c.662A>G, p.D221G | CA-ATS | S40/E10 |
| 8 | M | 42 | c.662A>G, p.D221G | 2V-CAD, PCI | S40/E10 |
| 9 | M | 41 | c.662A>G, p.D221G | - | S40/E10 |
| 10 | M | 68 | c.662A>G, p.D221G | 3V-CAD, PCI, CA-ATS | S40/E10 |
| 11 | M | 66 | c.682G>T, p.Q228X | Tx, 3V-CAD, PCI at 62, angina, CA-ATS | A40/E10 |
| 12 | F | 47 | c.1075C>T, p.Q359X | Tx, Ac, CA-ATS | S20/E10 |
| 13 | M | 42 | c.1211C>T, p.T404I | Ac | S40/E10 |
| 14 | F | 64 | c.1285G>A, p.V429M | Tx, Ac, CA-ATS | R10 |
| 15 | M | 65 | c.1415-1418dup, p.Q474HfsX42 | Tx, CA-ATS | A40 |
| 16 | M | 78 | c.1415-1418dup, p.Q474HfsX42 | Tx, Ac, 3V-CAD, CABG at 71 | S40/E10 |
| 17 | F | 45 | c.1474G>A, p.D492N | - | S20/E10 |
| 18 | M | 42 | c.1567G>A, p.V523M | MI at 28, 2V-CAD, PCI at 37 | A40 |
| 19 | F | 58 | c.1567G>A, p.V523M | CA-ATS | S20/E10 |
| 20 | M | 54 | c.1570G>A, p.V524M | 2V-CAD, angina | S60/E10 |
| 21 | M | 40 | c.1586G>A, p.G529D | CA-ATS | R10/E10 |
| 22 | M | 48 | c.1646G>A, p.G549D | Tx, Ac, CA-ATS | R20 |
| 23 | M | 57 | c.1735G>T, p.D579Y | Tx, 3V-CAD, CABG at 36, PCI at 47 | S60/E10 |
| 24 | F | 68 | c.1735G>T, p.D579Y | Tx, angina, CA-ATS | S40/E10 |
| 25 | F | 58 | c.1735G>T, p.D579Y | Tx, 2V-CAD, PCI at 50 | R20/E10 |
| 26 | M | 38 | c.1735G>T, p.D579Y | - | R10 |
| 27 | M | 36 | c.1735G>T, p.D579Y | Tx | S20/E10 |
| 28 | F | 62 | c.1735G>T, p.D579Y | Tx, Ac, CA-ATS | S40/E10 |
| 29 | M | 68 | c.2308C>T, p.Q770X | Tx, Ac, silent ischemia, CA-ATS | S40/E10 |
| 30 | M | 52 | c.2390 –1G>A | MI, 3V-CAD, CABG at 36 | S40/E10 |

The *LDLR* gene mutations were designated following the Human Genome Variation Society, version 2011 (the numerical series of codons includes the sequence of the signal peptide, [www.hgvs.org/mutnomen](http://www.hgvs.org/mutnomen)); Tx: tendinous xanthomatosis; Ac: arcus corneal; 1V, 2V, 3V-CAD: 1, 2, 3-vessel coronary artery disease; MI: myocardial infarction; PCI: percutaneous coronary intervention**;** CABG: coronary artery bypass graft; CA-ATS: carotid atherosclerosis; S: simvastatin; A: atorvastatin; R: rosuvastatin; E: ezetimibe (the number after the letter indicates the dose in mg/day).
